# Sodium Alginate–Starch Capsules for Enhanced Stability of Metformin in Simulated Gastrointestinal Fluids

**DOI:** 10.3390/biomimetics9110716

**Published:** 2024-11-20

**Authors:** Roxana Gheorghita, Ioan-Ovidiu Sirbu, Andrei Lobiuc, Mihai Covasa

**Affiliations:** 1 Department of Biochemistry, Victor Babes University of Medicine and Pharmacy Timisoara, 300041 Timisoara, Romania; roxana.puscaselu@usm.ro (R.G.); ovidiu.sirbu@umft.ro (I.-O.S.); 2 The Department of Biological and Morphofunctional Sciences, College of Medicine and Biological Science, Stefan cel Mare University of Suceava, 720229 Suceava, Romania; mcovasa@usm.ro; 3 Center for Complex Network Science, Victor Babes University of Medicine and Pharmacy Timisoara, 300041 Timisoara, Romania

**Keywords:** biopolymer encapsulation, swelling ratio, drug delivery, controlled release, diabetes

## Abstract

The use of biopolymers in pharmaceuticals is well established, particularly for encapsulating biologically active compounds due to their beneficial properties. Alginate, widely recognized for its excellent encapsulation abilities, is the most commonly used biopolymer, while starch, typically known as insoluble dietary fiber, also serves as an effective agent for trapping and protecting compounds during processing, storage, and gastrointestinal transit. Sodium alginate–starch capsules with varying compositions were analyzed to develop metformin hydrochloride (MET) containing capsules with adequate physicochemical properties. In vitro testing with simulated gastrointestinal fluids showed that after 1 h, capsules with equal amounts of alginate and starch had a higher swelling ratio and better drug release behavior, despite lower MET entrapment efficiency compared to other formulations. Microstructural analysis revealed stability in simulated gastric fluids and solubility in simulated intestinal fluids, key factors in drug development. The results suggest that these biopolymeric compositions are highly resistant to gastric fluids and minimally soluble in the intestines, making them suitable for extended drug release. This research evaluates key technological parameters of a cost-effective encapsulation method for the controlled release of active substances, providing a versatile solution for pharmaceutical and biomedical applications.

## 1. Introduction

The on-demand delivery of innovative drugs or food supplements remains a significant challenge due to the degradation of both active ingredients and delivery vehicles during digestion and metabolism. Key properties for formulating effective drug delivery systems include bioavailability, biodegradability, and precise control over drug content. One approach to regulating drug loading is by utilizing pure drugs or bioactive substances [1]. In drug delivery, biodegradable polymers are widely used, as they break down into non-toxic components within the body and offer advantages such as non-allergenicity and non-immunogenicity [2]. Films and coatings have been applied in the food, pharmaceutical, and biomedical fields for enhanced delivery and protection of active compounds.

Drug delivery is a multidisciplinary field requiring knowledge from biochemistry, pharmaceuticals, medicine, and engineering to optimize the therapeutic effects of pharmaceutical formulas or biologically active compounds [3]. Techniques such as extrusion, emulsion, spray-drying, and layer-by-layer encapsulation have revolutionized the field by enabling the encapsulation of active substances within biopolymeric materials. One of the key properties of these biopolymeric encapsulations is their ability to remain stable in specific environments, such as gastric fluid, while allowing the release of compounds in others, like the intestinal environment. This adaptability, based on biopolymer composition and encapsulation methods, enables their use in a wide range of applications and makes them suitable for all consumers, without concerns about allergies, or dietary, ethical, or religious restrictions. Studies have shown that encapsulating active compounds in natural polymers is an effective method for preserving their stability and viability. This approach has been successfully applied to probiotics [4,5,6], vitamins [7,8], polyphenols [9,10], and drugs [11,12].

Alginate, known for its excellent encapsulation properties, has been extensively used in the development of pharmaceuticals and biomedical systems. Approved by the US Food and Drug Administration (FDA) for consumption in *quantum statis* doses [13], alginate remains one of the most researched biopolymers for drug delivery, despite the availability of various other biopolymers, such as proteins, lipids, polysaccharides, and polypeptides. Its ability to form two distinct types of gels, an acid gel at low pH and an ionotropic gel at higher pH, differentiates it from neutral molecules and enhances its application in the pharmaceutical field. Alginate’s excellent mucoadhesive properties, biocompatibility, and anti-inflammatory and antioxidant effects make it highly suitable for active drug delivery applications [14].

While not as commonly used as alginate in newer formulations, starch is a cheap, versatile, and easy-to-process compound with non-irritating and non-toxic properties. It is widely used in the pharmaceutical industry for tablets and capsules as a diluent, disintegrant, binder, and lubricant [15]. Starch is one of the most abundant biopolymers on Earth, sourced from both conventional (corn, wheat, and potato) and unconventional sources (sago palm, quinoa, and young bamboo culm) [16]. Starch can be modified to meet the requirements for drug carriers and targeted release systems, offering benefits such as swelling capacity, mucoadhesive properties, extended shelf-life, and protection of drugs from extreme conditions, such as temperature fluctuations, pH changes, oxidation, and other ingredients [17]. The use of alginate and starch in products designed for intestinal release is supported by the presence of enzymes, such as protease, amylase, and pancreatic lipase, which contribute to the hydrolysis of the biopolymeric matrix and the release of bioactive compounds that are absorbed in the duodenum. Unabsorbed compounds are subsequently fermented by the gut microbiota [18]. Similar behavior has been observed in studies using alginate and protein-based capsules, which dissolve primarily in simulated gastric fluids [19]. In contrast, protein-based biopolymers can be utilized to develop products for gastric release, whereas polysaccharides are predominantly used for encapsulating drugs in biopolymeric matrices.

Metformin hydrochloride (MET), one of the most commonly prescribed medications for type II diabetes, is available in various film-coated tablet formulations that contain excipients like copovidone, povidone, titanium dioxide, lactose, and magnesium stearate. However, metformin has low oral bioavailability due to its poor permeability [20]. The advantages and disadvantages of previous encapsulating procedures used for MET are presented in Table 1.

This study aimed to explore the encapsulation of metformin hydrochloride (MET) in the sodium alginate and wheat starch matrices, using varied biopolymer concentrations to achieve capsules with reduced diameters. Our goal was to establish a straightforward production process that eliminates the need for specialized conditions and is easily scalable for industrial applications. Notably, our study distinguishes itself by incorporating a significantly higher starch ratio (17%) compared to existing studies, which typically employ lower starch levels (around 2% [34]). This increased starch proportion is expected to enhance cost efficiency, offering a novel approach to encapsulation that addresses economic feasibility directly. Also, unlike most studies focusing on sodium alginate combined with biopolymers like chitosan or carboxymethyl cellulose for improved encapsulation efficiency, our design relies solely on sodium alginate and starch, without additional compounds such as carrageenan [35], gelatin [36], or oils [37]. Furthermore, the choice of wheat starch, as opposed to less common or modified starch sources (e.g., tapioca [38]), reduces production complexity, avoiding supplementary processing steps [39,40]. Our previous research identified wheat starch as particularly suitable for forming compact matrices [41], supporting its use in this simplified formulation. This study, therefore, evaluates the underexplored potential of sodium alginate and starch biopolymer matrices for MET encapsulation. Alginate’s established drug encapsulation properties, alongside the economic and functional benefits of starch, such as swelling ability and adaptability in tablet formation, provide a promising foundation for developing targeted and controlled release formulations for active compounds

## 2. Materials and Methods

### 2.1. Materials

Metformin hydrochloride (Gedeon Richter Romania) was purchased from a Romanian pharmacy. Sodium alginate from brown algae, medium viscosity (≥2000 cP, 2% (25 °C)), unmodified starch from wheat, ≤0.3% protein content, calcium chloride anhydrous, with 94% assay, simulated gastric fluids without enzyme, with pH 1.1–1.3 (25 °C, after dilution), phosphate-buffered saline (PBS), pH 7.2–7.6, and distilled water were used in this study. These reagents, of analytical grade, were sourced from Sigma Aldrich, Romanian branch. A metformin hydrochloride film-coated capsule served as the control sample. The control tablet contains 1000 mg of metformin hydrochloride and coating based on microcrystalline cellulose, povidone K30, sodium lauryl sulfate, corn starch, talc, colloidal silicon dioxide, and magnesium stearate. The removal of the coating was performed by running water washing.

### 2.2. Methods

#### 2.2.1. Capsules’ Development

Capsules were produced using the extrusion method with a Caviar box system, following the procedure outlined by Nayak et al. [42], with slight modifications. The process began with the solubilization of the biopolymers.

**Figure 1:**
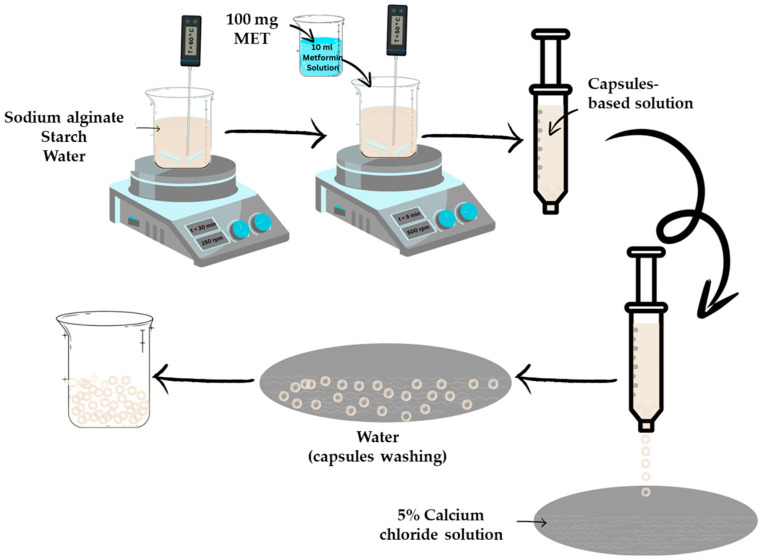
Flow diagram of the biopolymer-based capsule preparation process: The biopolymers and water are homogenized under stirring at 60 °C for 30 min. Subsequently, the MET solution is added, and the mixture is stirred for an additional 5 min. The resulting biopolymeric solution is then used for capsule formation.

Sodium alginate and starch were mixed in various proportions (as shown in Table 2) and solubilized in 150 mL of distilled water for 30 min at 60 ± 5 °C under continuous stirring (150 rpm), following the flow diagram in Figure 1. Once the biopolymer solution was prepared, the temperature was reduced by 5 °C. Simultaneously, 10 mL of metformin solution was prepared by dissolving 100 g of MET in water. The MET solution was then added to the biopolymer mixture and homogenized for 5 min at 500 rpm, maintaining the temperature at 50 ± 5 °C.

The solution was loaded into the Caviar-box system and dropped into a 5% CaCl_2_ cross-linking solution using a 96-gauge system (0.1 mm nozzle diameter). The capsules were maintained in the solution for 10 min to allow coating formation. After the capsules were developed, they were rinsed with fresh water to remove any excess solution and stored under refrigerated conditions (4 ± 2 °C) until further testing.

#### 2.2.2. Testing Methods

*Determination of particle size*. The diameters of 20 capsules were measured using a digital Yato micrometer (Shanghai, China) with an accuracy of 0.001. The results were expressed in micrometers (µm) as the mean ± SD of all readings.

*Evaluation of swelling ratio*. For this evaluation, 100 mg of capsules were immersed in a PBS solution. At different time intervals (1, 2, 3, 4, 5, 6, 8, and 24 h), the capsules were removed, placed on filter paper to eliminate excess water, and weighed. The swelling ratio was calculated using Equation (1).

(1) $$Sweeling\;ratio=\frac{Weight\;of\;particles\;after\;swelling-Weight\;of\;fresh\;particles\;}{Weight\;of\;fresh\;particles}\times 100$$

These tests were conducted only for the capsules containing MET (A–C). The production yield of the beads was calculated using Equation (2), following the method described by Lal et al. [43]:

(2) $$Production\;yield=\frac{weight\;of\;the\;dried\;beads}{weight\;of\;the\;drug\;taken+total\;polymer\;weight}\times 100$$

For drug loading evaluation, the total weight of the beads was measured immediately after the development process, and the results were calculated using Equation (3).

(3) $$Drug\;loading=\frac{Weight\;of\;the\;drug\;in\;the\;beads}{Weight\;of\;the\;beads}\times 100$$

**Figure 2:**
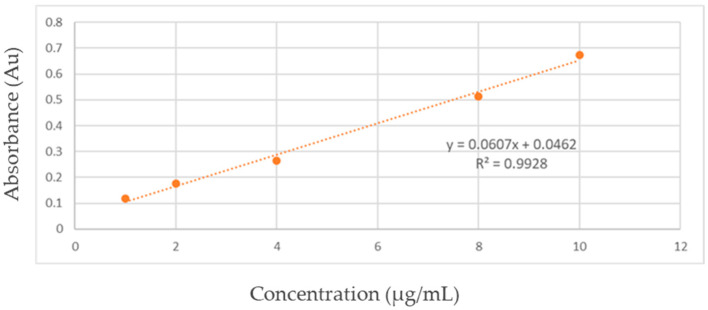
Calibration curve for dissolution study of MET 100 mg tested; y = absorbance; x = concentration in µg/mL.

*Calibration curve, encapsulation efficiency and in vitro drug release*. A spectrophotometric method was used to evaluate the MET-containing capsules (A–C). For the calibration curve, 100 mg of MET was dissolved in a lab jar and made up to 100 mL with distilled water. From this stock solution, 1 mL was taken and diluted to 10 mL. Aliquots of 0.2, 0.4, 0.6, 0.8, and 1 mL were transferred to standard flasks and diluted with PBS to 10 mL. The final concentrations were 2, 4, 6, 8, and 10 µg/mL. The absorbance of the samples was measured at 233 nm, and the calibration curve was obtained (Figure 2).

A linear regression equation was used to calculate the percentage of MET release at 0.5, 1, 2, 4, and 8 h. For encapsulation efficiency evaluation, 100 mg of capsules were crushed and dissolved in 10 mL of PBS buffer solution (pH 7.4). The suspension was transferred to a glass jar, and PBS was added to reach a final volume of 100 mL. This solution was maintained at room temperature for 24 h, with intermittent stirring. Afterward, the suspension was filtered, and the absorbance at 233 nm was measured using a calibration curve (Figure 2). The results were calculated according to Equation (4).

(4) $$Encapsulation\;efficiency=\frac{Actual\;loading}{Theoretical\;loading}\times 100$$

The in vitro release of the encapsulated drug was evaluated following the method described by Meligi et al. [44], with some modifications. Specifically, 100 mg of capsules were weighed and immersed in 3 mL of simulated gastric fluid (SGF, pH 1.2), stirred at 100 rpm, and maintained at 37 ± 2 °C for 2 h. Afterward, the capsules were filtered and transferred to 60 mL of fresh PBS solution (pH 7.4) to simulate the intestinal medium. At specific time intervals (0.5, 1, 2, 4, and 8 h), aliquots of 4 mL were withdrawn, and the absorbance at 233 nm was measured. The total volume in the flasks was replenished with fresh PBS solution up to 60 mL after each sampling.

*Surface morphology analysis*. The capsules were tested under several conditions using an optical microscope, using 40× magnification. To observe any changes that may occur during exposure to gastric and/or intestinal conditions and to identify the optimal composition for a targeted and controlled release system, capsules were immersed and maintained in simulated gastric and intestinal fluids. The analysis was conducted on particles tested in different conditions: fresh capsules (A1–F1), after 1 h maintenance in simulated gastric fluid (A2–F2), after 1 h maintenance in simulated intestinal media (A3–F3), and after 1 h in simulated gastric fluid, followed by 1 h in simulated intestinal media (A4–F4).

### 2.3. Statistical Analyses

One-way analysis of variance (ANOVA) was used to compare diameter, encapsulation efficiency, drug loading, and production yield across composition types (A–F). Two-way ANOVA was performed on swelling ratio (SR), with compositional type and time as factors. ANOVAs were followed by a post hoc Tukey test to determine significant differences. Correlation analyses between these physical characteristics were also performed. Results are presented as means ± standard deviation, with a *p*-value of <0.05 considered statistically significant. Statistical analyses were carried out using OriginPro Academic 2024 (OriginLab Corporation, USA, sourced from Romanian distributor).

## 3. Results and Discussion

The encapsulation of active ingredients in biopolymeric matrices has been demonstrated in various studies [45]. However, most research has focused on biopolymers such as sodium alginate, agar, and chitosan, with much less attention given to starch—a cheaper and easily processable alternative that does not require special preparation or equipment. Our results highlight the feasibility of using wheat starch and sodium alginate to develop biopolymeric capsules for prolonged and targeted release.

**Figure 3:**
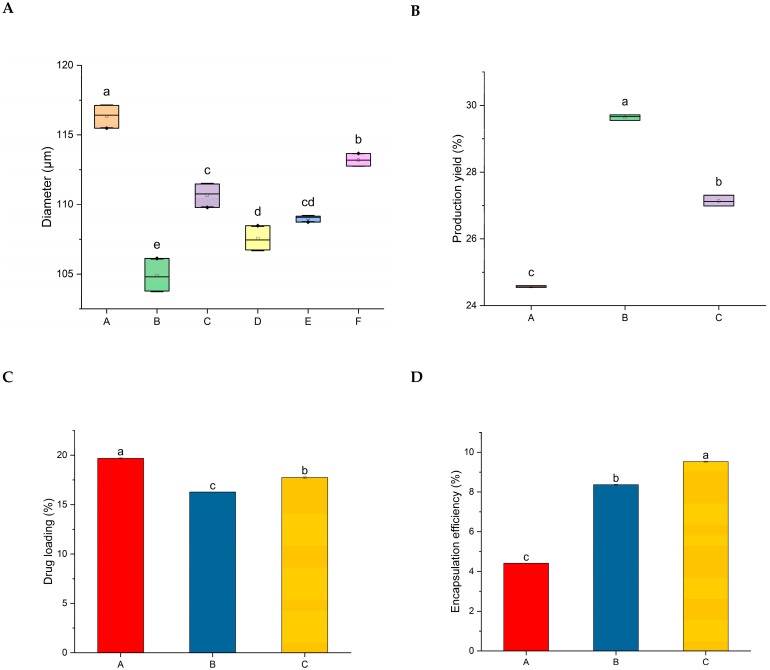
The diameter (**A**), production yield (**B**), drug loading (**C**), and encapsulation efficiency (**D**) of MET-containing capsules. A–E represent capsule formulations, according to Table 2. (**A**,**B**) Boxes represent the 25th–75th percentiles, with small squares indicating the mean ± SD. Values with differing letters are statistically different (*p* < 0.05). Bars are means ± SD.

The method we used for capsule production ensured uniform size ranges, with minimal variation (SD < 0.9%). Capsules with a higher sodium alginate content (Capsules A) had the largest diameter, measuring 116.34 µm. Most composition types were significantly different from one another; however, there was no statistical difference between C and E (110.67 ± 0.69 µm vs. 108.99 ± 0.18 µm) or between D and E (107.56 ± 0.71 µm vs. 108.99 ± 0.18 µm). Capsule diameters fell within a narrow range, from 104 to 116 µm, suggesting that the addition of MET to the capsules’ matrix did not exert any influence on this parameter (Figure 3A).

In their study, Talebian et al. [46] suggested that extrusion is one of the most effective methods for producing small, uniform spherical particles (5–500 µm) and is widely used for active ingredient delivery. Capsule size plays a crucial role in the release profile as well. Typically, smaller microcapsules exhibit faster release kinetics due to their higher surface-to-volume ratio [47]. For example, 3.5 mm beads quickly pass from the stomach to the intestine, whereas larger capsules (~12 mm) remain in the stomach longer and are only released when bolus viscosity increases [48]. For alginate-based capsules, lower solution viscosity facilitates development using the extrusion method, producing smaller-diameter capsules that resist bead deformation [49]. Given that our capsules size did not exceed 117 µm, their retention in the gastric environment is unlikely, supporting their potential for prolonged and targeted release potential.

The size of alginate-based capsules is particularly significant, as research has indicated that larger diameters are more prone to degradation in the gastric environment. For instance, using medium-viscosity alginate can result in capsule structures with pores that promote fluid absorption and solubilization. Gómez-Mascaraque et al. found that capsules with diameters larger than 380 ± 2 µm were more susceptible to faster disintegration [50]. Based on our findings, the small particle size and low swelling ratio enhance the potential of using this type of coating for MET encapsulation.

The production yields of the three formulations tested show statistically significant differences. As shown in Figure 3B, formulation B achieved the highest production yield (29.64 ± 0.13%), while formulation A had the lowest (24.56 ± 0.22%). These results indicate that adding starch to the capsule composition contributed to an increase in production yield (Figure 3B). Drug loading was affected by the formulation composition. The formulation with a higher sodium alginate content (A) had the highest drug loading (19.68 ± 0.46%), followed by composition C, which had an equal ratio of alginate to starch, with a drug loading of 17.74 ± 0.64% (Figure 3C). Finally, the encapsulation efficiency was significantly affected by the composition of the formulations tested. Composition C, containing equal amounts of sodium alginate and starch, had the highest encapsulation efficiency (9.52 ± 0.20%), compared to B (8.37 ± 0.20%) and A (4.42 ± 0.40%) (Figure 3D).

Our data show strong correlations between the formulations used and key parameters, including capsule size, drug loading, encapsulation efficiency, and production yield. The two-tailed test showed a negative correlation between capsule diameter and encapsulation efficiency (−0.72, *p* = 0.027) as well as between diameter and production yield (−0.98, *p* < 0.0001). A strong positive correlation was observed between diameter and drug loading (0.98, *p* < 0.0001). Additionally, there was a significant negative correlation between encapsulation efficiency and drug loading (−0.79, *p* = 0.02) and a strong positive correlation between drug loading and production yield (0.99, *p* < 0.0001).

The production yield, drug loading, and encapsulation efficiency in our study were considerably lower compared to the results obtained by Nayak et al., who developed alginate-starch capsules with aceclofenac [42]. In their study, the mean particle diameters were larger than those in our study (1.45–1.69 mm vs. 105–116 µm), which may account for the higher entrapment efficiency observed. Furthermore, the in vitro drug release profile showed 20% drug solubilization in acidic gastric conditions and more than 50% after 8 h of immersion. Smaller capsule diameters enhance resistance in the gastric environment, enabling the capsules to dissolve and release their contents in the intestinal environment. Consistent with the existing literature, capsules within the 100–150 micrometer range had excellent suitability as encapsulation systems for active ingredients. Our results showed a maximum MET release of 5.203% in the sample with the highest alginate content, and 6.323% in the sample with equal amounts of alginate and starch. Thus, MET was not released in sufficient quantities, even after 8 h under simulated gastrointestinal conditions. Thus, these formulations may be suitable for the early phase of diabetes mellitus treatment, where lower doses of metformin are typically required. During this stage, initiating therapy with a lower dose of MET is recommended to minimize gastrointestinal side effects. Conventional MET tablets are frequently associated with gastrointestinal issues, including diarrhea, vomiting, abdominal pain, bloating, and heartburn [51,52]. Increasing the content of starch and sodium alginate has demonstrated the potential for promoting controlled release [42].

**Figure 4:**
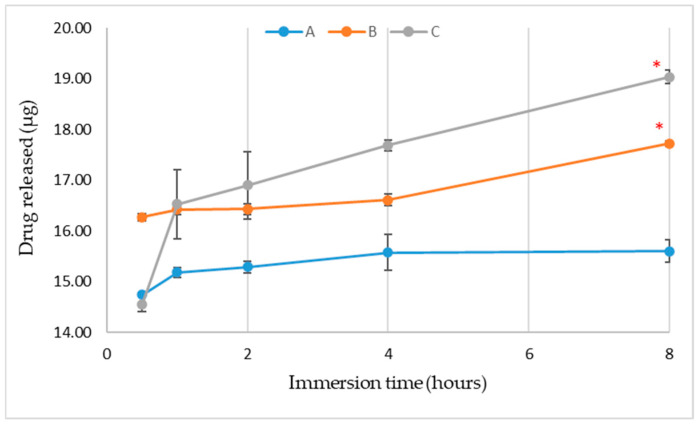
In vitro time-course profile of MET (100 mg) release. (A, 2500 mg sodium alginate, 500 mg starch); B, 2000 mg sodium alginate, 1000 mg starch; C, 1500 mg sodium alginate, 1500 mg starch). * indicates statistical difference compared to 0.5 h, *p* < 0.05.

Some studies have suggested that higher sodium alginate content leads to better encapsulation efficiency, an effect attributed to enhanced gelation between CaCl₂ and sodium alginate, providing more sites for ionic exchange [53]. Conversely, Cordoba et al. found that greater starch content improved encapsulation efficiency by approximately 10%, although it resulted in 85% release of the active substance in simulated gastric fluids. Using starch as a filler could be a solution, as it may delay the release of active compounds in gastric fluids [31]. In our study, encapsulation efficiency and drug loading were highest in composition A (with the greatest amount of sodium alginate) and lowest in composition C (with equal quantities of sodium alginate and starch) (Figure 4). This is consistent with the findings of Estevinho et al. who reported lower folic acid encapsulation efficiency with higher starch content. Their research also indicated that sodium alginate is an effective encapsulating agent for slower-release systems [54].

The different formulations in our study demonstrated that drug release can be controlled by adjusting the starch-to-alginate ratio. Over an 8 h period, drug release ranged from 14.5 µg at 0.5 h to 19 µg at 8 h (Figure 4), with a consistent trend of increasing MET release across all compositions. At 8 h, significant differences were observed among the formulations, with drug release inversely related to alginate content. The highest drug release at 8 h followed the order C > B > A, with types B and C releasing significantly more MET than type A (*p* < 0.05). The drug entrapment efficiency was higher when alginate was combined with chitosan as the encapsulating agent for MET [13]. However, the requirement to dissolve chitosan in acetic acid limits its industrial applicability due to potential effects on living systems and undesirable sensory characteristics. Starch, as one of the most cost-effective biopolymers, offers a more viable alternative for developing encapsulating matrices. Starch-based formulations are thus of greater interest compared to those involving chitosan. Another promising approach is hydrophobic ion pairing. Studies have shown that incorporating hydrophobic ion pairs into alginate beads can achieve over 80% encapsulation efficiency for MET. Another in vitro study showed only a 2.5% release of MET in gastric fluids after 24 h, with nearly complete release occurring within minutes in intestinal fluids. Without hydrophobic ion pairing, MET encapsulation efficiency was below 15% but increased to 60–100% with its inclusion [55]. This composition may be suitable for medications requiring rapid solubilization, such as analgesics and anti-inflammatory drugs. Our findings align with those of Andreopoulous and Tarantili who developed capsules containing salicylic acid, xanthan gum, and hydroxypropyl methylcellulose (HPMC). Their results showed complete dissolution of salicylic acid after 10 h for xanthan gum capsules, and approximately five days in HPMC capsules [56]. Another study observed that 89.6% of curcumin-alginate microspheres degraded in PBS by day 42 [53].

Sodium alginate and starch form stable capsules suitable for encapsulating bioactive substances via ionic cross-linking. Homayouni et al. demonstrated that combining alginate with starch provides effective protection for encapsulated bioactives [57]. This combination offers distinct advantages, including shielding sensitive ingredients from environmental factors such as pH fluctuations and enzymatic activity, thereby enabling controlled compound release across different regions of the gastrointestinal tract. In acidic environments (pH 1.5–3.5), the capsules exhibit stability, as observed in our study, maintaining protection of the active ingredients. The incorporation of starch into alginate capsules enhances structural integrity and modulates the release profile. Starch supports delayed or sustained release due to its gradual breakdown, especially in enzyme-rich environments, allowing for a more rapid dissolution at neutral pH (6–7.5). Given starch’s susceptibility to digestive enzymes, this composition promotes targeted release in the small intestine. While we did not conduct direct studies on release kinetics and mechanisms, the literature indicates that drug release from alginate-starch capsules typically follows a non-Fickian diffusion mechanism and that the power-law model does not apply to alginate capsules which dissolve within the first minute [34]. Non-Fickian pattern release that may result from capsule swelling and matrix solubilization is particularly valuable in pharmaceutical formulations as it enables the release profile of active substances to be adjusted, ensuring a steady therapeutic level over extended periods. This mechanism, combined with the structural benefits of the alginate-starch composition, highlights its potential for developing controlled-release pharmaceutical and nutraceutical formulations.

Our results are consistent with findings using fulvestrant, an FDA-approved drug for breast cancer treatment, which showed that only 38% of the drug encapsulated in alginate and chitosan was released after 120 days in PBS. Repeated on/off drug release behavior was observed using atomic force microscopy and ellipsometry, demonstrating the formula’s stability and controlled, sustained release profile for on-demand drug delivery. The study demonstrated that this formulation is highly suitable for applications such as implantable biosensors and antifouling coatings [58]. In agreement with this, our proposed formulations also show strong potential for similar applications, particularly in developing next-generation therapies for patients with type II diabetes. These patients benefit from continuous, controlled release of active substances to maintain stable blood glucose levels, and customized biopolymeric formulations could be tailored to meet their unique therapeutic needs.

**Figure 5:**
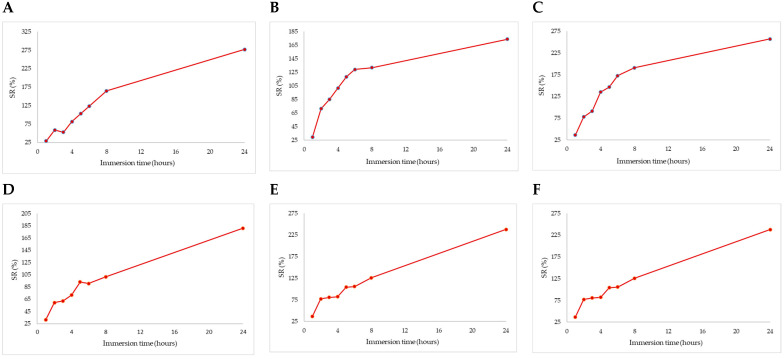
In vitro time-course swelling ratio of MET-loaded capsule formulations ((**A**–**F**), composition type).

SR was significantly increased during the 24 h time period for all compositional types (*p* < 0.0001) (Figure 5). During the first hour of immersion, the formulations containing MET exhibited the lowest swelling ratios, in the order A < B < C. The control, non-MET samples, displayed a similar pattern, with SR values in the order D < E < F. However, only formulations B and C showed statistically significant (*p* < 0.0001) differences in SR values compared to their controls (E and F, respectively). After 2 h, a similar trend was observed, with formulations B and C having statistically higher (*p* < 0.0001) SR values than the non-MET formulations E and F. By 4 h, this trend persisted, and all MET-containing formulations showed significantly higher (*p* < 0.0001) SR values than the non-MET types. At each subsequent measurement point, up to 24 h, all formulations continued to increase in swelling ratio with MET-containing capsules showing statistically higher (*p* < 0.0001) SR values compared to the non-MET controls. At 24 h, sample A, with the highest alginate content, showed approximately a 10-fold increase in SR (277.12% vs. 29.13%). Sample C, with a 1:1 starch-to-alginate ratio, had an almost eightfold increase (257.25% vs. 36.23%). Sample B, while following a similar trend in the first 6 h, showed a nearly sixfold increase in SR. This behavior may be attributed to starch-based capsules: as they swell and hydrate, the system’s viscosity increases, eventually reaching a saturation point where hydration slows [59]. Significant correlations were observed between drug release and SR at 2 h (r = 0.75, *p* = 0.01) and 4 h (r = 0.92, *p* = 0.0002). Thus, the addition of starch to the capsule composition promoted hydration, likely due to the hydroxyl groups (-OH) in the starch structure. These groups, with a high affinity for water, enable hydrogen bond formation, resulting in water absorption and capsule swelling. During the initial hydration phase, water molecules penetrate the amorphous regions of the starch granules, forming new hydrogen bonds that cause the capsules to swell and increase in diameter [59]. In contrast, the strength of capsules with a higher sodium alginate content can be attributed to Na+ ions, which participate in complexation and electrostatic interactions with the functional groups of the polymer matrix. A higher crosslink density results in a lower degree of swelling [60]. These findings highlight starch’s role in enhancing the swelling ratio. For example, capsules made from sodium alginate and chitosan showed a swelling ratio of only 71% [43]. The sustained increase in SR over an 8 h period, in the order A < B < C, suggests that the presence of MET promotes higher absorption. This effect can be attributed to MET’s hydrophilic nature, as it contains several polar groups, including amino and guanidine groups (three -NH3- and one -NH- group), which enhance solubility and facilitate water absorption into the capsule matrix. Additionally, the chemical and physical crosslinking points help maintain the 3D structure of the hydrogel in its swollen state [60].

**Figure 6:**
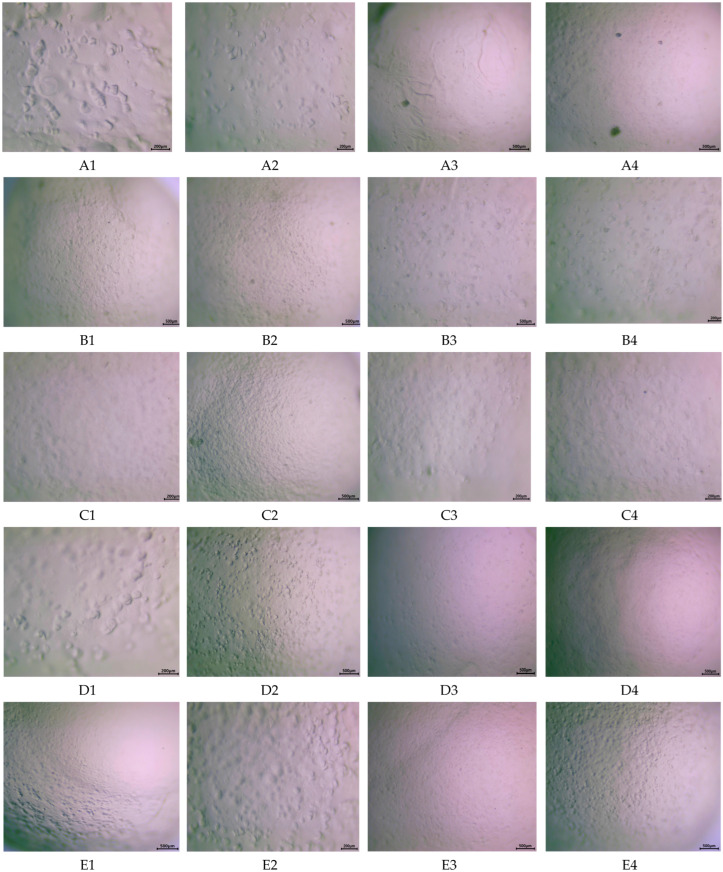

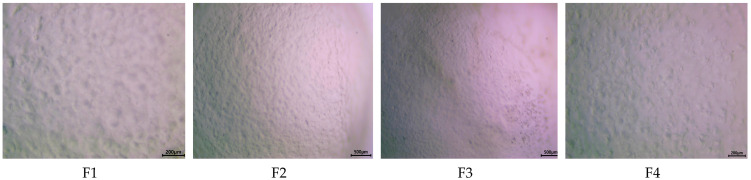
Microscopic images of capsules exposed to various gastric and intestinal conditions. (**A**–**F**) represents the sample composition and testing conditions: (1) fresh capsules; (2) 1 h in simulated gastric fluids; (3) 1 h in simulated intestinal media; (4) 1 h in simulated gastric fluids and 1 h in simulated gastric media.

As shown in Figure 6, the most notable structural changes appeared in formulations A and D, which contained a higher alginate concentration, independent of MET presence. The microstructure of these capsules exhibited significant alterations (A1 and D1) but remained stable in simulated gastric fluids, with minimal structural changes after one hour of exposure (A2 and D2). However, after one hour in intestinal fluids, the microstructure showed signs of solubilization. Interestingly, capsules without MET demonstrated lower stability in gastric fluids (A2 vs. D2, B2 vs. E2, and C2 vs. F2) and higher stability in intestinal media (A3 vs. D3, B3 vs. E3, and C3 vs. F3).

**Figure 7:**
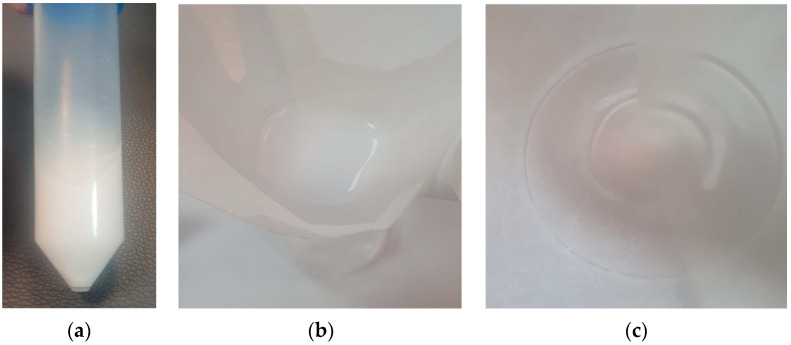
Behavior of the conventionally coated capsule in simulated gastric fluids: (**a**) complete dissolution of the tablet after one hour of immersion, as confirmed by the filtration of the resulting suspension (**b**,**c**).

The stability of the capsule coating in the gastric environment is a favorable characteristic, as it prevents dissolution in the acidic medium, preserving the active ingredients (MET in this case) for release in the intestinal environment. By contrast, the film-coated capsule used as the control sample dissolved almost completely after one hour of exposure to simulated gastric fluids (Figure 7).

The addition of sodium alginate to the biopolymeric matrix enhances capsule stability in both gastric and intestinal environments. Similar findings were reported by Narayani and Rao, who developed capsules using gelatin and sodium alginate. They observed that capsules with lower gelatin and higher alginate content remained intact during gastric retention (approximately 3 h) and only disintegrated upon reaching the ileocecal region of the intestine. In contrast, capsules with higher gelatin content disintegrated in the stomach within 15 min of ingestion [61]. The stabilizing effect of alginate is further supported by Zhang et al. [62], who found that capsules made solely from resistant starch completely disintegrated under both acidic gastric conditions (pH 1.2) and intestinal conditions (pH 6.8–7.2), suggesting these formulations may be more suited for rapid-release coatings. Additionally, studies have demonstrated that polysaccharides like alginate, carrageenan, and guar gum enhance capsule structure in acidic environments (pH 1.0) by preventing disintegration. Acid exposure strengthens cross-links between hydrocolloid chains, enhancing stability irrespective of the specific biopolymer ratios used [63].

## 4. Conclusions and Perspectives

Our studies yielded successful formulations of MET-loaded delivery systems using sodium alginate and wheat starch biopolymer-based capsules, demonstrating efficient MET encapsulation with extended-release capabilities. Capsule morphology and in vitro MET release tests confirmed that these biopolymeric compositions were highly resistant to gastric fluids and showed limited solubility in intestinal environments. Overall, capsules containing MET demonstrated greater swelling, and those with equal amounts of starch and alginate released the highest amount of MET after 8 h of dissolution.

Consequently, these compositions may be tested for encapsulating larger quantities of MET or other drugs requiring sustained release. While biodegradable and biocompatible polymers are generally considered safe, it remains important to assess their potential toxicity, immunogenicity, and long-term effects. Consequently, toxicological studies may be necessary to evaluate potential risks associated with biopolymeric capsules and identify any adverse effects. These risks may stem from the degree of extraction and possible impurities or chemical residues left after the extraction of biopolymers from sources such as brown algae. Additionally, the degradation products of biopolymers, including smaller molecules and byproducts, may be released under specific conditions (e.g., high temperatures, acidic, or enzymatic environments). Furthermore, regulatory guidelines for drug-embedded biopolymeric capsules should be updated to establish standards for the techniques and compositions involved, thus facilitating scalability for industrial production. Future research should focus on developing biopolymeric capsules through layer-by-layer technology and creating advanced drug delivery systems with alternative formulations, particularly those incorporating higher starch content to enhance functionality.

## Figures and Tables

**Table 1:** Development and encapsulation techniques of MET-loaded beads.

Technique	Formula	Advantages	Disadvantages	Reference
Extrusion	Alginate, tamarind seed and MET	The technique was straightforward, easily manageable, cost-effective, and reliable. Sustained in vitro drug release profile for 10 h.	Rough surface and presence of polymeric debris due to method of preparation.	[21]
	Sodium alginate and basil seed mucilage, and MET	Thermal stability of beads (up to 136 °C). Slower release behavior in acidic pH (1.2) and higher at pH 7.4.	Porous structure, with cavities in internal regions.	[22]
	Starch, alginate, and MET	Excellent mucoadhesive properties and a significant hypoglycemic effect in alloxan-induced diabetic rats over an extended period following oral administration. High drug encapsulation efficiency and good mucoadhesivity with biological membrane.	Not reported.	[23]
Emulsion	Alginate gel beads with MET	Being a cationic drug, MET’s release could be retarded due to interaction with alginate. Due to the oil entrapment technique, even a drug that is highly soluble in water can be delayed in the stomach.	The concentration of the oil influenced the pore size of the oil-entrapped beads. The higher the concentration of oil in the composition, the more the morphology of the capsules was affected: when the capsules were dried, they became uneven and lost their original spherical shape.	[24]
	Alginate and MET	Had hypoglycemic at low doses of alginate-metformin beads (approximately three times greater than that of pure metformin: 46.8 mg/kg vs. 150 mg/kg). Increased bioavailability in gastrointestinal tract.	Not reported.	[25]
	Polylactic acid and MET	Low encapsulation efficiency at high doses of MET (up to 50 mg).	The release profile was higher in gastric fluids than in intestinal conditions. Non-homogenous size distribution. The need for an extra stabilizer.	[26]
Spray- drying	MET nanoparticles	The drug maintained its effects for an extended period. A rapid technique which improved the dry powder MET tablet’s characteristics.	The use of toxic solvents, drug loading, and encapsulation efficiency are not high.	[27]
	Alginate, gelatin and MET	Improved bioavailability due to nano-scale diameter of particles and high surface area. Control of particle size and shape.	Long time of method processing and use of toxic reagents.	[28]
	Alginate, chitosan and MET	Excellent mucoadhesive properties, high drug loading capacity, and extended release of metformin hydrochloride.	Not reported.	[29]
Layer-by-layer	Sodium alginate, methyl cellulose sodium, and MET	The release kinetics can be controlled by modifying the concentration of the solution used for the layers, leading to coatings of different thicknesses.	Initial evaluation of the synergy among the biopolymers used. Potential unwanted interactions between the composition of each layer.	[30]
	Chitosan, pectin and MET			[31]
	Gellan and MET	100% MET release within 3 h (regular tablets in 1 h).	The maximum urinary excretion rate (dAU/dt) for MT was 36.55 ± 7.15 mg/h after 2.49 h post-administration. The commercial tablets achieved maximum rates of 19.36 ± 4.60 mg/h and 20.47 ± 5.61 mg/h at 5.67 and 7.17 h after administration, respectively.	[32]
Chitosomal and niosomal dispersion	Sodium alginate, chitosan, and MET	Notable enhancement in the hypoglycemic effect of MTF when administered as chitosomal and niosomal dispersion.	Almost 70% of the drug was released in 30 min in simulated gastric fluids.	[33]

**Table 2:** Formulation of capsules-forming solution.

Composition Type	Sodium Alginate, mg	Starch, mg	MET, mg
A	2500	500	100
B	2000	1000	100
C	1500	1500	100
D	2500	500	-
E	2000	1000	-
F	1500	1500	-

## Data Availability

The original contributions presented in this study are included in the article. Further inquiries can be directed to the corresponding author(s).
